# Selective Androgen Receptor Modulators Combined with Treadmill Exercise Have No Bone Benefit in Healthy Adult Rats

**DOI:** 10.3390/ph16091249

**Published:** 2023-09-05

**Authors:** Marina Komrakova, Arndt Friedrich Schilling, Wolfgang Lehmann, Veselin Vasilev, Katerina Georgieva, Fanka Gerginska, Nikolay Boyadjiev, Slavi Delchev

**Affiliations:** 1 Department of Trauma Surgery, Orthopaedics and Plastic Surgery, University Medical Center Goettingen, 37075 Goettingen, Germany; 2 Department of Physiology, Medical University of Plovdiv, 4002 Plovdiv, Bulgaria; 3 Department of Anatomy, Histology and Embryology, Medical University of Plovdiv, 4002 Plovdiv, Bulgaria

**Keywords:** ostarine, ligandrol, treadmill exercise, bone tissue, healthy rat model

## Abstract

The effects of combination treatments using the selective androgen receptor modulators (SARMs) ostarine (OST) or ligandrol (LIG) with treadmill exercise (TE) were studied in healthy adult rats. Fifteen-week-old male Wistar rats were divided into groups (*n* = 10/group). Experiment 1 consisted of (1) Control group: sedentary rats receiving vehicle; (2) OST: sedentary rats receiving OST; (3) TE: training rats receiving vehicle; (4) OST + TE: training rats receiving OST. Experiment 2 consisted of (1) LIG: sedentary group receiving LIG; (2) LIG + TE: training group receiving LIG. The TE regime was as follows: 25 m/min, 5° elevation, 40 min, five times/week, and the sedentary regime was 5 min, three times/week. OST and LIG were administered subcutaneously (0.4 mg/kg body weight/day, five times/week). After eight weeks, bone samples underwent microcomputed tomographical, biomechanical, histological, and ashing analyses. All the treatments had weak effects on the bone structure without affecting bone biomechanics. The OST + TE improved bone structure, while the LIG + TE had unfavorable effects. In serum, OST, OST + TE, and LIG + TE altered cholesterol and lipoprotein levels; TE did not change the serum parameters. The SARM treatments had no clear bone benefit, and the serum effects can be considered as side effects. TE represents a safe treatment. Because SARMs are increasingly applied in gyms along with physical activities, attention should be paid to possible side effects.

## 1. Introduction

Physical exercise is one of the primary factors associated with bone health, likely due to muscle–bone interactions. Bone mass and structure adapt to the mechanical stress caused by the muscles. The loss of musculoskeletal tissue with aging leads to the development of osteoporosis and other disorders [1,2]. Physical activity is therefore recommended as a proven means for osteoporosis prevention and rehabilitation [3], as exercise interventions during childhood and adulthood are supposed to mitigate age-related bone loss [1].

Further, sex hormones are essential for the maintenance of bone mass. In males, osteoporosis occurs primarily due to diminished testicular testosterone production. Testosterone supplementation can be used as a treatment for this condition, but it has severe negative side effects [4,5]. Therefore, the application of nonsteroidal selective androgen receptor modulators (SARMs) has been proposed for osteoporosis therapy based on their selective anabolic effects on musculoskeletal tissue [6,7]. Ostarine (OST, enobosarm, S-22, MK-2866, or GTx-024) and ligandrol (LIG, LGD-4033, or VK5211) are nonsteroidal SARMs that bind to the androgen receptor (AR) with some tissue selectivity and cannot be converted to dihydrotestosterone and estrogen. They are thought to have fewer side effects than testosterone [7,8,9,10]. OST and LIG are still being investigated in clinical trials and are not currently approved for human use in any country [9,11,12,13,14]. Nevertheless, they are sold on the internet and are used by bodybuilders and other athletes. They are included in the list of prohibited anabolic agents by the World Anti-Doping Agency (https://www.wada-ama.org/en/prohibited-list?q=anabolic, S1 anabolic agents, accessed on 18 August 2023).

There is an expectation that exercise and other interventions, such as SARM use during young adult life, can help to sustain healthy bone tissue and attenuate its age-related deterioration. In the present study, we investigated whether a combination treatment of treadmill exercise (TE) with OST or LIG, which are increasingly utilized in gyms to enhance muscle mass and strength [15], could affect healthy bone tissue in adult rats. This healthy mature rat model mimics the situation in healthy adult bodybuilders who use SARMs along with physical activity.

## 2. Results

Body weight did not differ between the groups at the beginning or at the end of the study in both experiments (Table 1).

The biomechanical properties, stiffness, and Fmax of all the bones studied did not differ significantly between the groups in both experiments (Table 1).

In ashing analysis of the femur, the mineral content, magnesium, calcium, and phosphate, as well as the calcium–phosphate ratio, were not different between the groups in both experiments (Table 1). Data on calcium and phosphate contents are not shown.

Micro-CT 3D analysis revealed no differences in bone parameters in Experiment 1, whereas total BMD and BV/TV were lower in the LIG + TE group than in the LIG group in the distal femur in Experiment 2 (Table 2).

**Figure 1:**
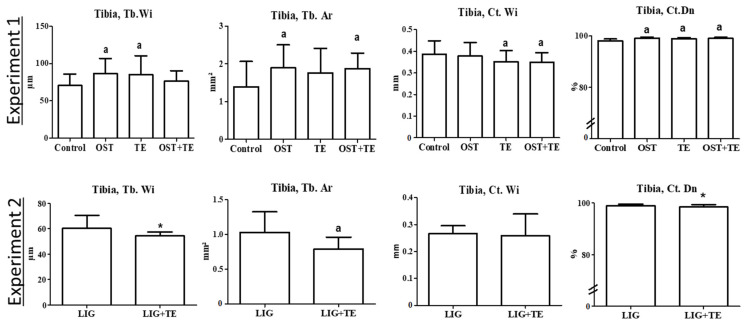
Bone parameters of proximal tibia in the micro-CT 2D analysis. Ostarine (OST), ligandrol (LIG), and treadmill treatments (TE); Ct: cortical, Tb: trabecular, Wi: width, Dn: density, Ar: area. Experiment 1: (a) different from Control group (*p* < 0.05, Tukey test). Experiment 2: (*) different from LIG group (*p* < 0.05, *t*-test).

Micro-CT 2D analysis of the distal femur showed reduced Ct.Dn in the TE group and reduced Ct.Wi in the OST + TE group compared with the Control and OST groups, whereas N.Nd was higher in the TE group than in the OST + TE group in Experiment 1 (Table 3). Ct.Wi was also thinner in the TE group than in the Control group in the distal femur. In Experiment 2, Tb.Wi and Ct.Wi were thinner in the LIG + TE than in the LIG group in the distal femur (Table 3). In the proximal femur, trabecular parameters were higher in the OST + TE group than in the Control group, and the Tb.Wi was thicker in this group than in the OST group in Experiment 1 (Table 3). In L3, N.Nd was higher in the OST + TE group compared with the Control group in Experiment 1. In Experiment 2, N.Nd and Ct.Dn were lower in the LIG + TE group than in the LIG group (Table 3). Most of the tibia parameters were different after treatments compared with the Control group in Experiment 1 (Figure 1). Tb.Wi was enhanced in the OST and TE groups, Tb.Ar was larger in the OST and OST + TE groups, and Ct.Dn was higher in all three treated groups. Ct.Wi was thinner in the TE and OST + TE groups than in the Control group (Figure 1). In Experiment 2, tibia parameters (Tb.Wi, Tb.Ar, and Ct.Dn) were lower in the LIG + TE group than in the LIG group (Figure 1).

**Figure 2:**
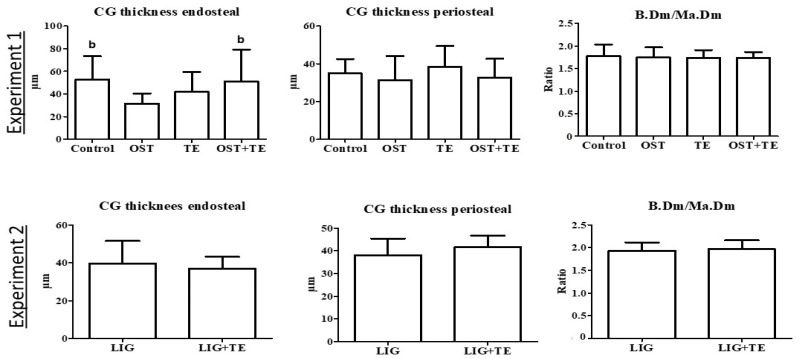
Bone parameters in the histological analysis. Ostarine (OST), ligandrol (LIG), treadmill treatments (TE), and calcein green staining (CG). Experiment 1: (b) different from OST group (*p* < 0.05, Tukey test). Experiment 2: differences were not significant (*p* > 0.05, *t*-test).

In the histological analysis of the tibia, the endosteal apposition rate was higher in the Control and OST + TE groups than in the OST group in Experiment 1 (Figure 2). In Experiment 2, no differences were observed (Figure 2).

## 3. Discussion

Regular physical activity is important for building and maintaining healthy bone tissue in children, adolescents, and older people [1]. The SARMs OST and LIG have been reported to have a favorable effect on the musculoskeletal system [9,11,12,13,14]. Although they are not currently approved for use in any country, bodybuilders use them to improve muscle mass and strength. The effect of physical activity and SARMs on bone tissue in adolescents is not well understood. In the present study, we investigated whether combination treatments of OST or LIG with TE could affect healthy bone tissue in an adult rat model. Additionally, the effects of OST and TE alone were evaluated.

All treatments exerted only a weak bone response in healthy adult rats, determined mainly through 2D analyses of bone structure. The effect was favorable in the trabecular bone, whereas in the cortical bone, which is less metabolically active, it was ambiguous. The effect could be ordered as TE = OS < OS + TE in Experiment 1 and LIG > LIG + TE in Experiment 2.

The OST treatment had only minor effects on bone tissue, enhancing the trabecular width and area as well as the cortical density based on 2D micro-CT analysis of the proximal tibia, and decreasing the apposition rate of the cortical bone as determined with a histological analysis of the distal tibia. No effect was observed on other skeletal sites. There are reported benefits of OST therapy for osteoporosis and sarcopenia in tumor cachexia, stress incontinence, and breast cancer [12,13,16]. Age and the hormonal status of treated animals appear to have an impact on the effects of the SARMs on bone tissue. In the present study, the animals were healthy and relatively young. The weak effect on bone in this condition contrasts with the anabolic effect observed in older, hormone-deficient male rats [16]. Osteo-anabolic SARMs such as OST affect bone tissue by stimulating osteoblast activity, which increases the mineralization rate [17]. The osteo-anabolic effect of SARMs through androgen receptors (ARs) has been reported previously [13], and the presence of endogenous testosterone and dihydrotestosterone, which are also bound to the ARs [17], apparently dampened the effect of OST on bone tissue in the present study. A possible explanation could be that the compounds replace the missing function of the endogenous hormones but cannot add anything in their presence. As the use of SARMs is continuing to increase in the fitness and bodybuilding communities, a few clinical studies have investigated their pharmacokinetic profiles and identified potential adverse effects [9,15,18]. However, studies on the effects of OST on bone tissue in healthy adult individuals are lacking.

Similar to OST, TE treatment increased the trabecular width and cortical density in the proximal tibia, whereas the cortical density in the distal femur and the cortical width in the proximal tibia were decreased. Most previous studies have reported a favorable effect of treadmill training on bone tissue [19,20,21,22,23]. Various treadmill training regimes have been applied, and the bone response has been shown to vary based on age, sex, and skeletal site. Further, hormonal status was reported to contribute to differences in musculoskeletal sensitivity to mechanical stimulation, which was more effective in 5-month-old estrogen-deficient rats than in healthy ones [24]. In young 6-week-old female rats, seven and eleven weeks of TE increased the femoral length and tibial bone mineral content but did not alter the lumbar bone parameters [22]. Yeh et al. [19] found that in 14-month-old female rats, bone mineral content and BMD were increased in the tibia, whereas in the vertebrae, only BMD was increased after nine-week TE. Oxlund et al. [20] reported a doubling of the mineralizing surface in the femur after 73-day TE but no impact on bone mechanical properties in 18-month-old female rats. Wu et al. [23] found that eight-week TE increased the BMD in the femur but not in the vertebral body of 24-month-old male rats. In healthy adult 13-week-old male rats, eight weeks of systemic submaximal training, similar to that applied in the present study, had an anabolic effect on some trabecular bone parameters in the femur, enhanced the osteogenic potential, inhibited the adipogenic differentiation of bone mesenchymal stem cells derived from the treated animals, and stimulated β-catenin protein expression in vivo and gene expression in vitro [21]. However, the effect on cortical bone was not reported in that study [21]. Thus, the previous study as well as the current study show stronger effects of TE on the bone of the hindlimbs than of the vertebrae. This could be explained by the active role of hindlimbs in quadrupedal locomotion during TE and by the greater activity of the leg muscles than the back muscles, which in turn stimulates the tibia and the femur more than the spine. Further, trabecular bone is more metabolically active than cortical bone [25], and thus the anabolic effect of the treatments was mostly seen in trabecular bone, whereas the effect was smaller and less clear in cortical bone.

The combined treatment with OST and TE changed more bone parameters than single treatments, but only in 2D micro-CT and histological analyses. The bone biomechanical properties and 3D bone parameters were not changed. All trabecular parameters were higher under the combination therapy than in the Control and OST groups in the proximal femur and trabecular area, and the cortical density in the proximal tibia and endosteal bone formation in the distal tibia were both increased. The cortical width decreased in the proximal tibia and distal femur. Both OST and TE have anabolic effects on bone cells directly and also indirectly through the muscle tissue [17,21]. OST activates bone formation by binding to ARs on osteocytes and osteoblasts [13] and increases the mass and weight of muscles, affecting bone tissue through mechanical loading and muscle–bone crosstalk [26]. Muscle contractions during TE also affect bones mechanically, and physical activity plays a role in bone–muscle crosstalk [27]. Further, bone tissue is sensitive to mechanical signals through mechanoreceptors and piezo-mechanosensitive channels in bone cells [28]. This could explain the synergistic effects of OST and TE on bone tissue.

In contrast to the OST + TE treatment, the combination of LIG with TE showed a negative effect on bone structural parameters in comparison with LIG alone. The changes in bone were detected not only in 2D analyses, as in Experiment 1, but also in the micro-CT 3D analysis. Total BMD, BV/TV, and trabecular and cortical width were reduced in the distal femur. Additionally, the number of trabecular nodes and the cortical density in L3, as well as the trabecular width, trabecular area, and cortical density in the proximal tibia, were also decreased compared with the single LIG treatment. Similar to OST, LIG has been reported to stimulate ARs and has an anabolic effect on the musculoskeletal system [9,29]. LIG showed a favorable effect on bone structural parameters without changing biomechanical properties in an ovariectomized rat model [29]. In a clinical phase 1 study of 76 adult men, a dose-dependent increase in muscle mass was observed without significant adverse side effects over 21 days with daily doses of 0.1, 0.3, and 2 mg [9]. However, a recent case report described the development of severe drug-induced liver injury after a two-week administration of LIG (10 mg/day) in a 32-year-old man [30]. Another case report showed that a five-week coadministration of LIG (10 mg/day) and a growth hormone secretagogue (MK-677) in a 25-year-old man increased body mass, lean body mass, fat mass, serum lipid levels, liver enzymes, intramuscular testosterone, and dihydrotestosterone, while bone mineral density, bone mineral content, and the intramuscular expression of ARs were decreased [14]. This effect on bone is concordant with our results. Cardaci et al. [14] suggested that changes in bone tissue might be attributable to an LIG-mediated decrease in endogenous testosterone levels and, consequently, less conversion to estrogen. It is known that estrogens play a major role in bone growth and in the regulation of bone turnover in both sexes [31]. In the present study, although the differences between the LIG and LIG + TE treatments were observed solely in structural bone parameters while biomechanical properties were not affected, LIG + TE was found to be unfavorable for bone structure. Therefore, further investigations should be focused on combined therapies, as the administration of the SARMs OST and LIG along with physical activity is becoming more popular to enhance aesthetics, muscular strength, and athletic performance despite a lack of data on their utility and safety-related aspects [14].

The subtle effects observed in bone structure were not sufficient to change the biomechanical parameters, mineral, calcium, magnesium, and phosphate contents in bone with either of the treatments. However, they could protect bone against osteoporosis (OST + TE) or promote osteoporotic changes (LIG + TE), as the bone micro-architecture is impaired during aging [2].

Analysis of serum parameters in our study revealed an enhanced level of cholesterol in the OST group. SARMs have been reported to decrease cholesterol levels in several previous studies [9,18,32]. In serum, the total cholesterol contains HDL, LDL, as well as triglycerides. However, none of these parameters were affected by OST in the present study. OST + TE elevated HDL levels in comparison with the Control group. High levels of HDL cholesterol are considered to be an indicator of a healthy cardiovascular system [33,34]. Further, the beneficial effects of regular physical activity on cholesterol levels have been well established [33]. Whereas a high level of HDL is considered healthy, a high level of LDL can increase the risk of cardiovascular complications [33,34]. In Experiment 2, the LDL level was higher in the LIG + TE group than in the LIG group. An unambiguous interpretation of this finding cannot be provided due to the absence of an untreated control group in this experiment and a lack of reference data for the rat model [35]. Another limitation of the present study is the lack of a baseline control at the beginning of both experiments. Therefore, it remains unknown whether LIG decreases LDH, as observed previously [9,18], or if the combination of LIG + TE enhances LDH. The TE single treatment did not change any of the serum parameters in healthy adult rats, and thus this treatment appears to be a safe option for healthy adult individuals.

Body weight was not changed by any of the treatments in this study. Similarly, there were no changes observed after OST or LIG treatments on the body weight of ovariectomized female rats [36]. However, in these rats, the effect of estrogen depletion was so strong that it could overlap the effect of the SARMs [36]. In contrast, the body weight of 8-month-old orchiectomized rats was increased under OST treatment [16]. In clinical studies, a relative increase in lean body mass was observed as a result of SARM treatments in patients with cachexia and sarcopenia (reviewed by Molfino et al.) [37]. Similar to the findings in bone, hormonal and health status seem to determine the body’s response to SARM treatments.

## 4. Materials and Methods

### 4.1. General Procedures

The animal study protocol was approved by the local regional government (Bulgarian Food Safety Agency (BFSA) license no. 294) prior to the study. Sample size was calculated using G-Power analysis (G*Power program, Heinrich-Heine-University, Düsseldorf, Germany) prior to the study. Bone mineral density of lumbar vertebral bodies from the previous study was used for calculations [16]. An F-test (analysis of variance) was applied to calculate the sample size for Experiment 1 and a *t*-test for Experiment 2 with a power (1-β error) of 80% and α-type I error of 0.05. Ten animals per group were considered sufficient to detect the possible effect of the treatments based on these calculations and our previous experience.

Healthy 15-week-old male Wistar rats (Vivarium at Bulgarian Academy of Sciences (BAS)) were used in the experiments. The health status of the animals was confirmed by a veterinarian prior to the study. The rats were housed individually and had free access to tap water and standard pelleted rodent diet (Amiko A Ltd., Belozem, Bulgaria) throughout the experiment. As training on the treadmill is a skill that must be learned by the experimental animals, before the beginning of the experiments, all rats ran on a treadmill made for small laboratory animals (EXER-3R-Treadmill, Columbus Instruments, Columbus, OH, USA) for 5 min a day, three times weekly, at a speed of 25 m/min, with a 5° elevation, for two weeks. This exercise duration and intensity does not induce adaptation [38]. Rats that refused to run were excluded from the experiments. In Experiment 1, 40 spontaneously running rats were divided into the following four groups (*n* = 10/group): (1) Control: sedentary group receiving vehicle treatment; (2) OST: sedentary group receiving OST; (3) TE: training group receiving vehicle; and (4) OST + TE: training group receiving OST. In Experiment 2, there were two groups of rats (*n* = 10/group): (1) LIG: sedentary group treated with LIG and (2) LIG + TE: training group receiving LIG.

The training rats were subjected to submaximal training on the treadmill for eight weeks. They ran at a speed of 25 m/min, with a 5° elevation, five days per week. On the first training day, the duration of the exercise was 20 min, and it was increased by 5 min every second day until reaching a duration of 40 min per day at the end of the first week. This exercise duration was maintained until the end of the experiments. The nontrained rats ran for 5 min, three days per week, at the same speed and track elevation [39]. One rat from the OST group was excluded due to a disease, and another rat from the TE group was also excluded because of injuries that did not allow it to perform the training schedule.

OST (Shanghai Biochempartner Co., Ltd., Shanghai, China) and LIG (Hölzel Diagnostika Handels GmbH, Cologne, Germany) were administered subcutaneously (s.c.) dissolved in polyethylene glycol 300 and dimethyl sulfoxide (80% and 20%, respectively) at a daily dose of 0.4 mg/kg body weight, five times per week [36]. The vehicle groups received only solvent.

On the fifth and first days before sampling, all rats were injected s.c. with calcein green fluorescent dye (10 mg/kg BW, Waldeck GmbH, Münster, Germany) to label newly built bone tissue [40].

After eight weeks of treatments, all rats were decapitated under i.p. ketamine (Medistar, Holzwickede, Germany) and xylazine (Riemser, Greifswald-Insel Riems, Germany) anesthesia (180 and 15 mg/kg BW, respectively). Decapitation was performed with a small animal guillotine (HUGO SACHS ELEC-TRONIC, March-Hugstetten, Germany), and serum and bone samples were immediately collected for further analysis. Bone samples (lumbar vertebral body 3 [L3], both tibiae and femora) were collected for biomechanical, microcomputed tomographical (micro-CT), histological, and ashing analyses. Statistical analysis was performed using a one-way analysis of variance and a Tukey post hoc test (*p* < 0.05) in Experiment 1 and a *t*-test in Experiment 2 (*p* < 0.05) with the aid of the GraphPad Prism program (Version 5.0, San Diego, CA, USA).

### 4.2. Serum Analyses

Freshly collected serum was analyzed within a few hours after sampling. The analyses of glucose, cholesterol, LDL, HDL, and triglycerides were conducted at the Department of Clinical Laboratory, Medical University Plovdiv (Plovdiv, Bulgaria), using a Konelab 60i clinical chemistry analyzer (Thermo Fisher Scientific, Vantaa, Finland) according to the manufacturer’s instructions.

### 4.3. Biomechanical Analysis

**Figure 3:**
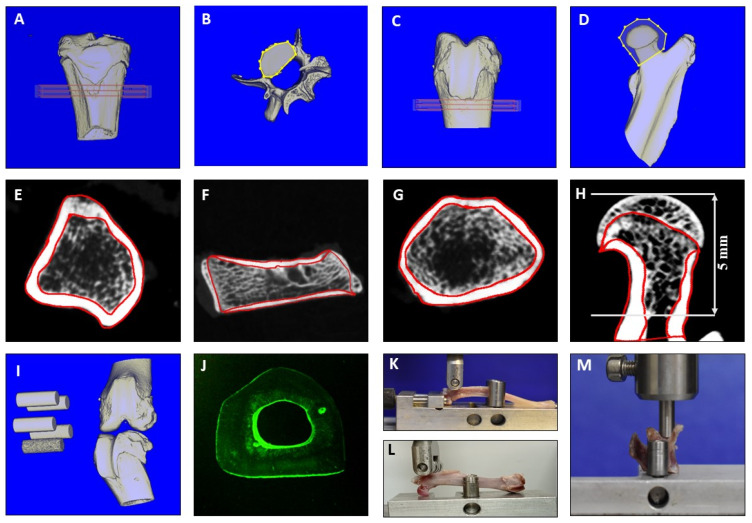
Micro-CT 3D analysis: (**A**) proximal tibia, (**B**) L3, (**C**) distal femur, (**D**) proximal femur, (**I**) 5 hydroxyapatite elements in each scan. Measurement frame in tibia and femur was: 1 × 10 × 10 mm^3^ shown in red (**A**,**C**). Corpus vertebrae in L3 (**B**) and femoral head in proximal femur bounded yellow were assessed. Micro-CT 2D analysis: (**E**) proximal tibia, (**F**) L3, (**G**) distal femur, (**H**) proximal femur. (**E**–**H**): red bounded regions of cortical and trabecular bone. (**J**) fluorescence analysis of tibia. Biomechanical analyses: (**K**) tibia, (**L**) femur, (**M**) L3. Samples from the Control group, Experiment 1.

Biomechanical parameters of the bone samples were assessed with a Zwick machine (145,660 Z020/TND; Ulm, Germany). The L3 was tested with compression testing (Figure 3M). It was fixed at the aluminum base, and the stamp was loaded to the vertebral body [16]. The femur and tibia were analyzed using a three-point bending test (Figure 3K,L). The femur was loaded to the trochanteric region, and the tibia to the proximal metaphysis, until they broke [16,41]. The stamp was loaded at 50 mm/min and stopped automatically by software (TestExpert, Zwick/Roell) when the applied force decreased more than 20 N for the femur and tibia and 10 N for L3. Stiffness (N/mm), the slope of the linear increase in the curve during elastic deformation, and the maximal force (Fmax, N) that the bone could withstand before it broke, were calculated using Microsoft Excel (Microsoft Office 2016) [16].

### 4.4. Micro-CT Analyses

Bone samples were scanned using a Quantum FX micro-CT (Caliper Sciences, Hopkinton, MA, USA) using the following scan protocol: 70 kilovoltage peak (kVp), 200 μA, 2 min exposure time, 360° rotation, 3600 projections, 20 × 20 mm^2^ field of view, 512-pixel matrix, and 40 × 40 × 40 μm^3^ effective voxel size [40]. Five hydroxyapatite elements of varying mineral densities were scanned with each bone to convert the data into bone mineral density (Figure 3I). The scans were analyzed using the Scry program (Scry v6.0 software, Kuchel and Sautter UG, Bad Teinach-Zavelstein, Germany) [42].

The region of interest in the femur was the femoral head and distal metaphysis (Figure 3C,D). The proximal metaphyseal part of the tibia was analyzed (Figure 3A). In L3, the corpus vertebra was separated and evaluated (Figure 3B). Standard thresholds for soft tissue, trabecular and cortical bone, bone tissue, and total tissue were set for the femur, tibia, and L3 [16,43]. The following three-dimensional (3D) bone parameters were measured according to ASBMR criteria: total bone mineral density (total BMD), cortical and trabecular densities (Ct.BMD and Tb.BMD), and bone volume fraction (BV/TV) [44]. Bone structure was analyzed using two-dimensional (2D) images created with the Scry program. Three images of the sagittal cut femoral head, distal metaphysis of the femur, proximal metaphysis of the tibia, and the vertebral body (Figure 3E–H) were analyzed using MetaMorph Basic Acquisition software (Leica Mikrosysteme Vertrieb GmbH, Wetzlar, Germany). The following parameters were measured: cortical density (Ct.Dn, % of bone tissue), trabecular density (Tb.Dn, %), number of trabecular nodes (N.Nd), trabecular width (Tb.Wi, µm), cortical width (Ct.Wi, mm), and trabecular area (Tb.Ar, mm^2^) [44,45].

### 4.5. Histological Analysis

The tibia samples were exposed to sequential ascending concentrations of ethanol and embedded in Technovit^®^ 9100 medium (Heraeus Kulzer GmbH, Wehrheim, Germany). Sections 150 µm in thickness were cut transversally 2 cm distal to the knee surface using a diamond saw microtome (Leica SP 1600, Leica Instruments GmbH, Nussloch, Germany). Three sections of the tibia were mounted with Eukitt medium (O. Kindler GmbH, Freiburg, Germany), digitalized using a digital camera (Leica DFC490) and a zoom stereo microscope (Leica DMRXE), and analyzed with the aid of the MetaMorph image analysis program (Leica, Bensheim, Germany) (Figure 3J). The following parameters were measured: the thickness of CG-stained new bone at the endosteal and periosteal sites of the cortical bone, marrow diameter (Ma.Dm), bone diameter (B.Dm), and the bone-to-marrow diameter ratio (B.Dm/Ma.Dm) [40].

### 4.6. Ashing Analysis

Femur samples were ashed in a muffle oven at 750 °C for 2 h. The bones were weighed before and after ashing to the nearest 0.000001 g. Mineral content was determined using the ash weight and expressed relative to the wet weight of each bone (%) [16]. Calcium and magnesium contents were assessed using an atomic absorption spectrometer (4100, PerkinElmer, Hamburg, Germany) according to the guidelines of the European Committee for Standardization (CEN) [46]. Orthophosphate content was determined using the colorimetric method (2030 Multilabel Reader Viktor X4, Perkin Elmer, Turku, Finland) according to CEN [47] guidelines.

## 5. Conclusions

In conclusion, all treatments evaluated in the present study had minimal effects on bone structure without affecting bone quality in healthy adult rats. Some trabecular bone parameters were increased under OST treatments. The combination of OST and TE had positive synergistic effects, whereas the combined treatment of LIG and TE affected bone structure unfavorably. In serum, OST and both combination therapies altered cholesterol or lipoprotein levels, which can be considered as side effects. The effect of TE on bone was also weak. Meanwhile, TE had no side effects on serum parameters and can be considered safe for healthy adult individuals, which is consistent with the general and scientific consensus. Because the administration of OST and LIG along with physical activity has become increasingly popular among healthy adult bodybuilders as an alternative to steroid hormones [14,15], they should be critically evaluated to reveal potential negative side effects.

## Figures and Tables

**Table 1:** Body weight, serum, biomechanical parameters, and ashing analyses of lumbar vertebral body 3 and femur (Experiments 1 and 2).

Parameters	Experiment 1				Experiment 2	
	Control *n* = 10	OST *n* = 9	TE *n* = 9	OST + TE *n* = 10	LIG *n* = 10	LIG + TE *n* = 10
	Mean ± SD	Mean ± SD	Mean ± SD	Mean ± SD	Mean ± SD	Mean ± SD
Initial body weight (g)	229 ± 21	228 ± 26	223 ± 14	230 ± 21	234 ± 18	233 ± 16
End body weight (g)	422 ± 43	419 ± 29	413 ± 38	442 ± 45	391 ± 23	377 ± 53
***Serum analyses (mmol/L)***						
Cholesterol	1.5 ± 0.2	1.9 ^a^ ± 0.2	1.7 ± 0.3	1.7 ± 0.2	2.0 ± 0.3	1.9 ± 0.5
Glucose	6.4 ± 2.0	5.0 ± 1.1	6.6 ± 1.4	5.2 ± 1.8	3.9 ± 12	3.3 ± 8
HDL	1.3 ± 0.2	1.5 ± 0.2	1.4 ± 0.2	**1.5 ^a^ ± 0.1**	1.5 ± 0.2	1.3 ± 0.3
LDL	0.3 ± 0.1	0.4 ± 0.1	0.4 ± 0.1	0.4 ± 0.1	0.2 ± 0.1	0.5 * ± 0.2
Triglyceride	0.4 ± 0.1	0.5 ± 0.2	0.5 ± 0.3	0.5 ± 0.2	0.6 ± 0.2	0.6 ± 0.3
***Biomechanical analyses Femur***						
Stiffness (N/mm)	308 ± 49	289 ± 51	244 ± 48	246 ± 52	222 ± 39	241 ± 80
Fmax (N)	169 ± 18	169 ± 20	157 ± 28	148 ± 24	139 ± 19	142 ± 19
***Tibia***						
Stiffness (N/mm)	106 ± 28	114 ± 41	104 ± 41	97 ± 33	128 ± 47	99 ± 18
Fmax (N)	56 ± 8	61 ± 11	59 ± 15	54 ± 5	70 ± 16	69 ± 22
***L3***						
Stiffness (N/mm)	281 ± 54	279 ± 31	277 ± 45	275 ± 61	258 ± 46	294 ± 81
Fmax (N)	209 ± 46	199 ± 31	194 ± 51	198 ± 23	211 ± 37	186 ± 63
***Ashing analysis, femur***						
Mineral content (%)	44 ± 2	42 ± 2	42 ± 2	42 ± 1	41 ± 2	41 ± 3
Mg^+^ (%)	0.77 ± 0.03	0.75 ± 0.02	0.77 ± 0.03	0.75 ± 0.01	0.76 ± 0.02	0.77 ± 0.03
Ca^2+^/PO_4_^3−^	1.66 ± 0.03	1.68 ± 0.06	1.73 ± 0.09	1.71 ± 0.06	1.35 ± 0.02	1.37 ±0.05

OST: ostarine, LIG: ligandrol, TE: treadmill treatments. Experiment 1: (a) different from Control group (*p* < 0.05, Tukey test). Experiment 2: (*) different from LIG group (*p* < 0.05, *t*-test). Significant differences are highlighted in bold.

**Table 2:** Micro-CT 3D analysis of bone (Experiments 1 and 2).

Parameters		Experiment 1			Experiment 2	
	Control *n* = 10	OST *n* = 9	TE *n* = 9	OST + TE *n* = 10	LIG *n* = 10	LIG + TE *n* = 10
	Mean ± SD	Mean ± SD	Mean ± SD	Mean ± SD	Mean ± SD	Mean ± SD
***Femur distal***						
Total BMD (g/cm^3^)	0.54 ± 0.05	0.58 ± 0.06	0.55 ± 0.07	0.52 ± 0.06	0.58 ± 0.05	0.50 * ± 0.07
BV/TV (%)	64 ± 8	70 ± 11	68 ± 8	65 ± 9	54 ± 8	44 * ± 9
Ct. BMD (g/cm^3^)	1.12 ± 0.04	1.13 ± 0.05	1.11 ± 0.03	1.11 ± 0.04	1.14 ± 0.02	1.13 ± 0.02
Tb. BMD (g/cm^3^)	0.57 ± 0.01	0.56 ± 0.02	0.56 ± 0.02	0.55 ± 0.01	0.65 ± 0.01	0.64 ± 0.01
***Femur proximal***						
Total BMD (g/cm^3^)	0.75 ± 0.03	0.70 ± 0.06	0.71 ± 0.04	0.72 ± 0.05	0.71 ± 0.05	0.67 ± 0.07
BV/TV (%)	73 ± 4	69 ± 7	58 ± 6	70 ± 6	70 ± 6	62 ± 11
Ct. BMD (g/cm^3^)	1.10 ± 0.03	1.12 ± 0.06	1.13 ± 0.05	1.12 ± 0.04	1.09 ± 0.02	1.09 ± 0.04
Tb. BMD (g/cm^3^)	0.77 ± 0.01	0.76 ± 0.01	0.75 ± 0.02	0.76 ± 0.02	0.76 ± 0.02	0.75 ± 0.02
***Tibia proximal***						
Total BMD (g/cm^3^)	0.36 ± 0.04	0.39 ± 0.04	0.38 ± 0.05	0.40 ± 0.04	0.46 ± 0.04	0.43 ± 0.07
BV/TV (%)	44 ± 7	50 ± 9	50 ± 5	51 ± 6	46 ± 6	42 ± 7
Ct. BMD (g/cm^3^)	1.03 ± 0.03	1.00 ± 0.04	1.00 ± 0.06	1.01 ± 0.07	1.12 ± 0.03	1.12 ± 0.05
Tb. BMD (g/cm^3^)	0.47 ± 0.01	0.46 ± 0.01	0.46 ± 0.02	0.46 ± 0.01	0.56 ± 0.01	0.57 ± 0.01
***L3 corpus vertebrae***						
Total BMD (g/cm^3^)	0.59 ± 0.01	0.57 ± 0.03	0.57 ± 0.03	0.58 ± 0.03	0.62 ± 0.04	0.57 ± 0.08
BV/TV (%)	74 ± 5	72 ± 5	73 ± 3	74 ± 2	68 ± 5	61 ± 12
Ct. BMD (g/cm^3^)	1.01 ± 0.01	1.02 ± 0.02	1.01 ± 0.01	1.02 ± 0.02	1.06 ± 0.02	1.05 ± 0.02
Tb. BMD (g/cm^3^)	0.60 ± 0.01	0.59 ± 0.01	0.59 ± 0.02	0.59 ± 0.01	0.65 ± 0.01	0.65 ± 0.02

OST: ostarine, LIG: ligandrol, TE: treadmill treatments, L3: lumbar vertebral body 3, BMD: bone mineral density, Ct: cortical, Tb: trabecular, BV/TV: bone volume fraction. Experiment 1: differences were not significant (*p* > 0.05, Tukey test). Experiment 2: (*) different from LIG group (*p* < 0.05, *t*-test). Significant differences are highlighted in bold.

**Table 3:** Micro-CT 2D analysis of bone (Experiments 1 and 2).

Parameters		Experiment 1			Experiment 2	
	Control n = 10	OST n = 9	TE n = 9	OST + TE n = 10	LIG n = 10	LIG + TE n = 10
	Mean ± SD	Mean ± SD	Mean ± SD	Mean ± SD	Mean ± SD	Mean ± SD
***Femur distal***						
N.Nd (n/mm^2^)	9.5 ± 2.4	9.5 ± 2.3	10.7 ± 1.3	**8.9 ^c^ ± 1.8**	9.1 ± 1.8	8.3 ± 1.7
Tb.Wi (µm)	87 ± 23	98 ± 24	94 ± 22	87 ± 18	87 ± 18	69 * ± 9
Tb.Dn (%)	21 ± 7	24 ± 8	24 ± 4	23 ± 6	22 ± 5	16 ± 4
Ct.Dn (%)	98 ± 1	98 ± 1	**97 ^ad^ ± 1**	98 ± 1	98 ± 1	98 ± 1
Ct.Wi (mm)	0.33 ± 0.04	0.34 ± 0.05	**0.31 ^b^ ± 0.05**	**0.30 ^ab^ ± 0.05**	0.30 ± 0.05	0.26 * ± 0.07
***Femur proximal***						
N.Nd (n/mm^2^)	4.2 ± 2.0	4.4 ± 1.1	4.8 ± 1.2	**5.6 ^a^ ± 1.8**	4.0 ± 1.2	3.8 ± 1.2
Tb.Wi (µm)	78 ± 13	82 ± 8	87 ± 17	**95 ^ab^ ± 14**	76 ± 10	78 ± 11
Tb.Dn (%)	27 ± 9	29 ± 4	31 ± 7	**34 ^a^ ± 7**	26 ± 4	26 ± 5
Ct.Dn (%)	96 ± 2	96 ± 1	97 ± 1	97 ± 1	97 ± 1	98 ± 1
***L3***						
N.Nd (n/mm^2^)	8.5 ± 2.0	9.0 ± 1.6	7.9 ± 1.5	**9.6 ^c^ ± 2.1**	9.6 ± 2.1	8.0 * ± 1.5
Tb.Wi (µm)	131 ± 31	122 ± 27	124 ± 39	108 ± 23	87 ± 17	85 ± 23
Tb.Dn (%)	30 ± 6	30 ± 5	30 ± 5	28 ± 5	24 ± 3	22 ± 8
Ct.Dn (%)	94 ± 2	93 ± 3	94 ± 2	94 ± 3	94 ± 3	92 * ± 3

OST: ostarine, LIG: ligandrol, TE: treadmill treatments, L3: lumbar vertebral body 3, Ct: cortical, Tb: trabecular, Wi: width, Dn: density, N.Nd: number of nodes. Experiment 1: (a) different from Control group, (b) different from OST group, (c) different from TE group, (d) different from OST + TE group (*p* < 0.05, Tukey test). Experiment 2: (*) different from LIG group (*p* < 0.05, *t*-test). Significant differences are highlighted in bold.

## Data Availability

Data is contained within the article.
